# Agrometeorological drought in the Romanian plain within the sector delimited by the valleys of the Olt and Buzău Rivers

**DOI:** 10.1186/s40201-014-0152-0

**Published:** 2014-12-27

**Authors:** Ovidiu Murărescu, George Murătoreanu, Mădălina Frînculeasa

**Affiliations:** Department of Geography, Valahia University of Târgoviște, Târgoviște, Romania

**Keywords:** Precipitation, Temperatures, Water climatic, Drought, Soil

## Abstract

**Background:**

The last few decades have recorded a high frequency of the meteorological drought phenomenon. Southern and south-eastern Romania make no exception, with such phenomena often occurring from July to November 2011, which brought about an agrometerological drought that lasted from the third decade of July to early December, with a slight improvement in October. This situation led to a decrease in soil water reserves, mainly in the first 20 cm, with a negative impact on agricultural crops and the following agricultural year as well.

**Findings:**

The methodology was based on a correlative analysis between the decadal rainfall quantities and the existing soil water reserve, during the interval between June and November 2011, for eight weather stations.

**Conclusion:**

The statistico-mathematical data analysis showed an intensification of the pedological drought phenomenon in September, with a slight improvement in October and an increase in November.

## Introduction

The increase of the meteorological drought phenomenon leads to the occurrence of the pedological drought and, hence, of aridity, combined with a high soil temperature and evapotranspiration. Such situations, according to statistical history, were recorded in 2000, 2001, 2007, 2011, 2012 and 2013.

**Figure 1:**
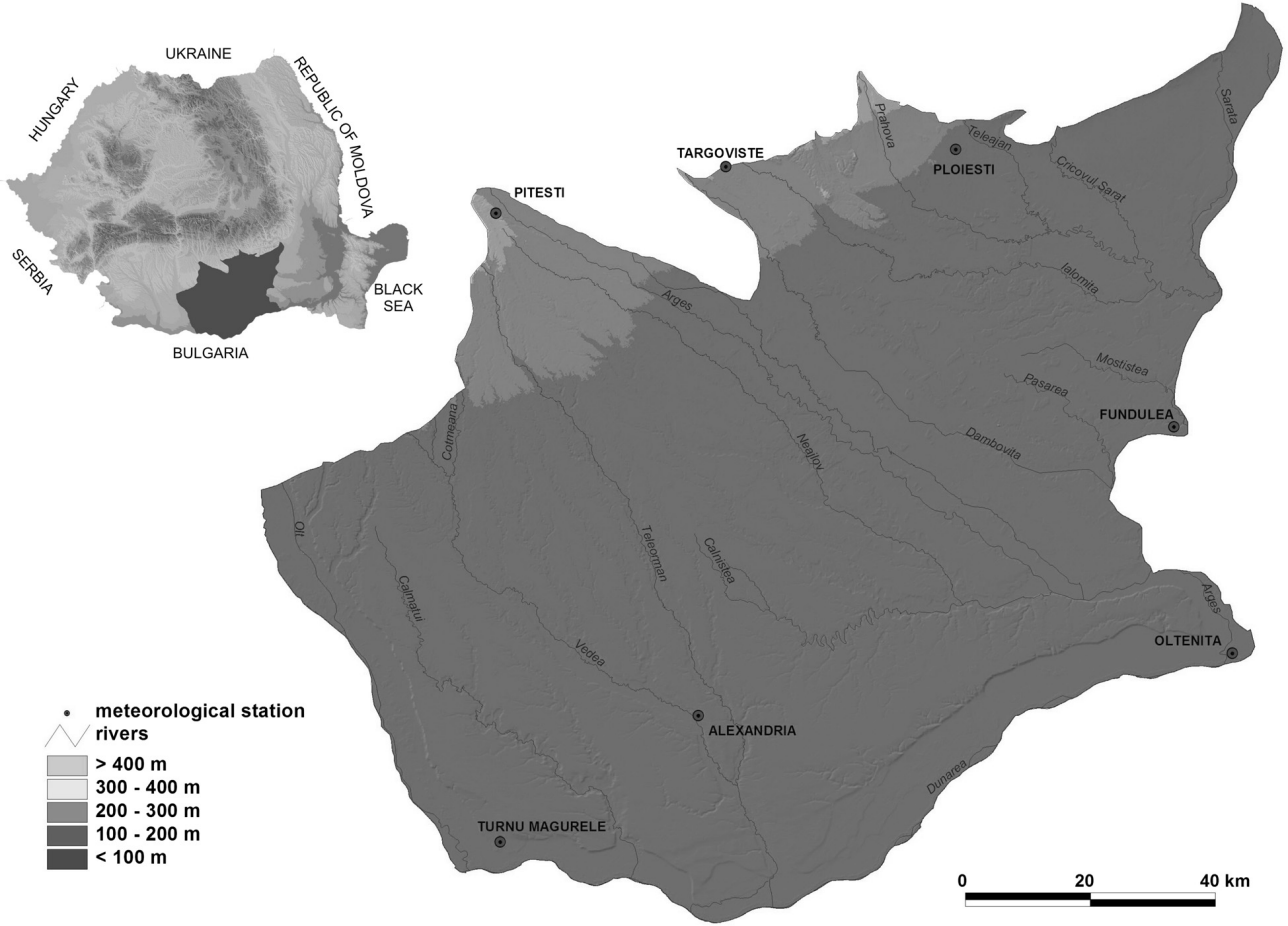
**The analysed geographical area.**

The analysed geographical area includes the Romanian Plain, more exactly the sector between the left bank of the Olt river – in the west – as far as its confluence with the Danube; north-eastern boundary is located on the right bank of the Buzău river; the northern is conventionally located north of Slatina, Valea Mare, Potcoava, then the contact between the Cotmeanu Piedmont and the 5th terrace of the Argeş river as far as north of Piteşti, stretching along the contact with the Outer Subcarpathian Curvature Hills; the southern boundary is located at the contact with the Danube valley, while the eastern overlaps the interference between the forest-steppe and the forest – the Argeş river meadow, from the confluence with the Dâmboviţa river to its flow into the Danube, then Dâmboviţa – Pasărea – Obârşia Mostiştei and Sărata – boundary with the Bărăgan Plain [1] (Figure 1).

There have been concerns regarding the analysis of the phenomena of aridity occurrence ever since early 20th century (Lang – 1920 – the rain factor; De Martonne – 1926 – the aridity index – etc.). In Romania, many climatological geographers have had similar preoccupations [2-19].

### Data and working methods

The present study does not aim to calculate the climatic water deficit (CWD) and the De Martonne aridity index (I_ar_), as these parameters have already been identified, and correlations between them have also been made [11,14,17], therefore the territorial map of the aridity index in Romania – mm/°C has been drawn as well [2].

This approach aims rather to identify and characterise the agrometeorological phenomena of hydric risk by taking into consideration parameters and critical thresholds during the intervals which are specific to phenological processes and phases. Such analyses can be performed decadally, bimonthly, monthly, seasonally, annually or permanently with a view to adopting negative effect prevention and reduction measures [20]. In this regard, we have conducted a correlative analysis between the decadal rainfall quantities and the existing soil water reserve, during the interval between June and November 2011, for eight weather stations (Piteşti, Ploieşti, Târgovişte, Turnu Măgurele, Alexandria, Oltenţia, Fundulea).

The soil moisture considered was determined from the surface to one-metre depth by 20-cm sections. The available moisture is calculated based on the volumetric weight of the soil stratum considered (Vw), existing moisture content (U) and the wilting point (WP), according to the following formula:

$$ \mathrm{A}\mathrm{W}\mathrm{C}=0.1 \times \mathrm{V}\mathrm{w} \times \left(\mathrm{U}\%\ \hbox{-}\ \mathrm{W}\mathrm{P}\right) \times \mathrm{h} $$

where AWC = available moisture, and h = depth.

## Findings

In the calendar year 2011, the annual mean temperature in Romania was 9.2°C, 0.3°C higher than the climatological normal, with positive deviations between 0.1°C (in March and May) and 2-6°C in September, but also with negative deviations in February, April, October and November (0.1°C – April up to 2.7°C in November) from the climatological normals.

The country average annual rainfall was 500.4 mm, 22% under the climatological normal, due to the deficits recorded in most months. The excess rainfall was in June-July, while the rest of the months were characterised by deficit, with negative deviations ranging between 10-97%. Months with deficit were March (40%), August (53%), September (72%) and November (97%). As a result, the year 2011 was characterised by low rainfall, with November the driest month, and by pedological drought installed differentially at regional level [21].

The statistico-mathematical data analysis of decadal rainfall quantities recorded at the eight agrometeorological stations located in the field region under study, correlated with the soil water reserve recorded in July-November 2011, showed an intensification of the pedological drought phenomenon in September, with a slight improvement in October and an increase in November.

The average available moisture (AWC) required for plant development, according to norms, is 400 at 20-cm depth, 820 at 40 cm and 1500 at 80 cm.

**Figure 2:**
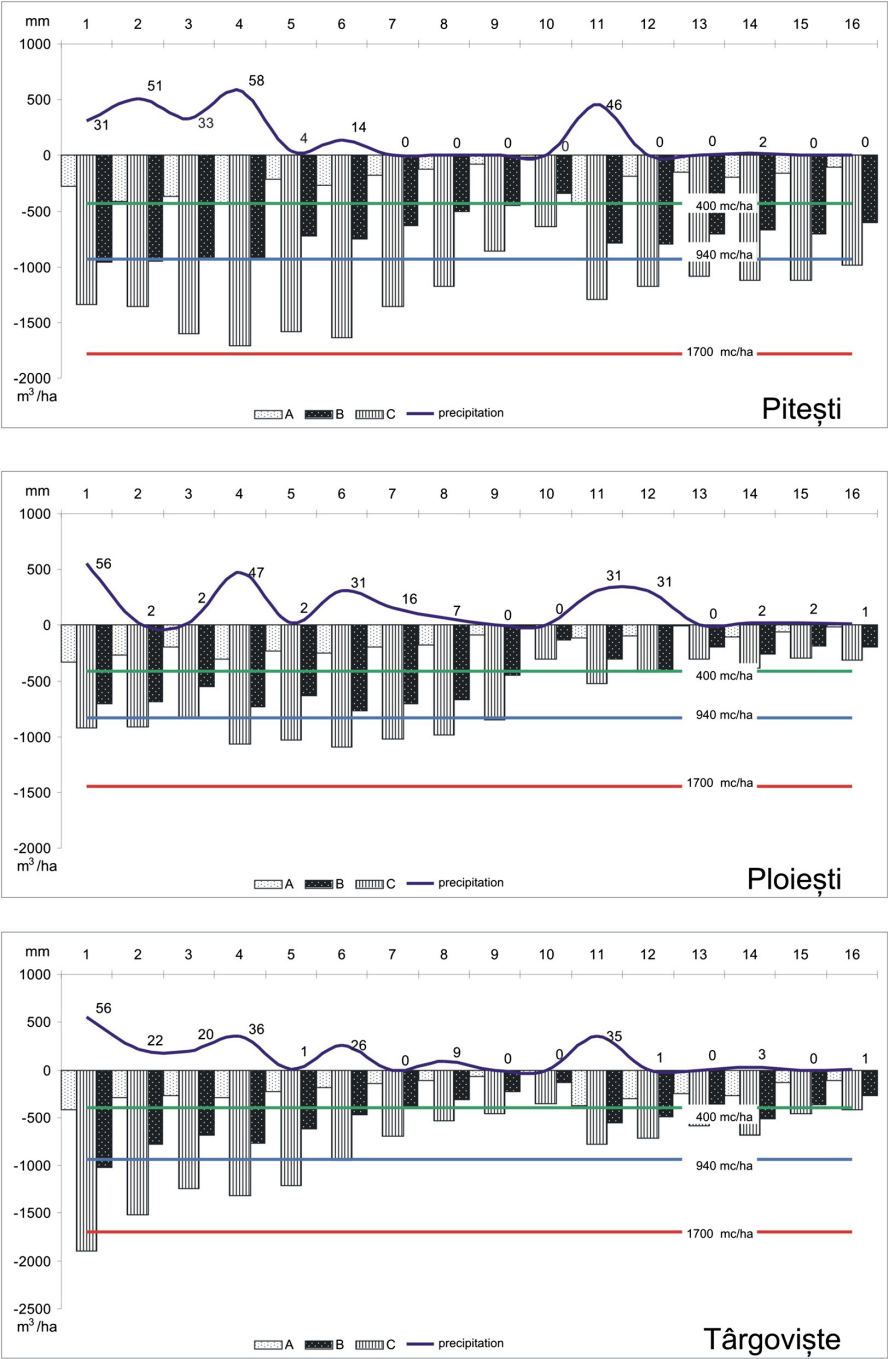
**The average available moisture (AWC) and the soil moisture reserve for the agrometeorological station situated in the north of the plain (data source: National Meteorology Administration).**

With the agrometeorological stations situated in the north of the plain (Piteşti, Târgovişte, Ploieşti), located on the 30 mm/°C isoline, according to the Romania aridity index map, deficits were observed in August (below 30 mm), September (0 mm) and November (5 mm). Under these conditions, the soil moisture reserve fell drastically, to values much below the normal averages (Figure 2).

**Figure 3:**
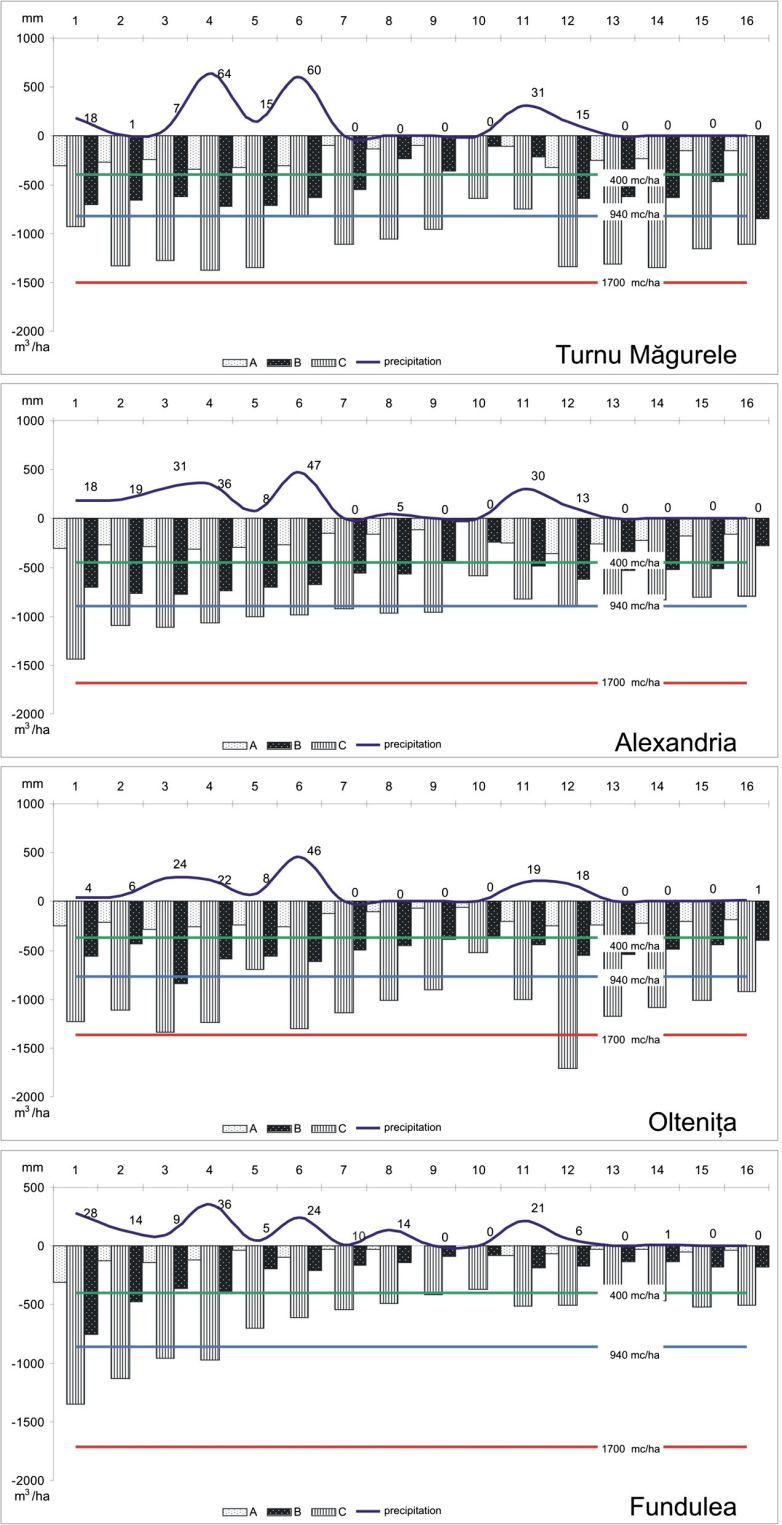
**The average available moisture (AWC) and the soil moisture reserve for the agrometeorological station situated in the south of the plain (data source: National Meteorology Administration).**

As regards the agrometeorological stations located on the 20 mm/°C isoline, according to the same map (Turnu Măgurele, Alexandria, Fundulea and Oltenţia and Buzău), the situation is similar in terms of precipitations, but with a slight extension between the decadal intervals towards the months of July and October (Figure 3).

## Conclusions

The results of the study have emphasised the consequences of an agrometeorological phenomenon of risk, with negative effects mainly on summer-autumn agricultural crops, leading to a decrease of the agricultural production, but also with significant implications on the following agricultural year (2012–2013), due to inability to perform specific agro-technical works or, if they had been carried out, they had to be redone because seeding had been compromised.

## Abbreviations

AWC: Available water capacity
